# Loss of MAPK-activated protein kinase 2 enables potent dendritic cell-driven anti-tumour T cell response

**DOI:** 10.1038/s41598-017-12208-7

**Published:** 2017-09-18

**Authors:** Klara Soukup, Angela Halfmann, Barbara Dillinger, Fiona Poyer, Katharina Martin, Bernadette Blauensteiner, Maximilian Kauer, Mario Kuttke, Gernot Schabbauer, Alexander M. Dohnal

**Affiliations:** 1 Tumour Immunology, St. Anna Kinderkrebsforschung, Children’s Cancer Research Institute, Vienna, Austria; 2 Bioinformatics, St. Anna Kinderkrebsforschung, Children’s Cancer Research Institute, Vienna, Austria; 3 Institute for Physiology, Centre for Physiology and Pharmacology, Medical University of Vienna, Vienna, Austria; 4 Present Address: Department of Fundamental Oncology, Ludwig Institute for Cancer Research, University of Lausanne, Lausanne, Switzerland; 5 APEIRON Biologics AG, Vienna, Austria

## Abstract

Maintaining dendritic cells (DC) in a state of dysfunction represents a key mechanism by which tumour cells evade recognition and elimination by the immune system. Limited knowledge about the intracellular mediators of DC dysfunction restricts success of therapies aimed at reactivating a DC-driven anti-tumour immune response. Using a cell type-specific murine knock-out model, we have identified MAPK-activated protein kinase 2 (MK2) as a major guardian of a suppressive DC phenotype in the melanoma tumour microenvironment. MK2 deletion in CD11c^+^ cells led to an expansion of stimulatory CD103^+^ DCs, mounting a potent CD8^+^ T cell response that resulted in elimination of highly aggressive B16-F10 tumours upon toll-like receptor (TLR) activation in the presence of tumour antigen. Moreover, tumour infiltration by suppressive myeloid cells was strongly diminished. These insights into the regulation of DC functionality reveal MK2 as a targetable pathway for DC-centred immunomodulatory cancer therapies.

## Introduction

The role of myeloid cells in promoting tumour progression by contributing to an immunosuppressive microenvironment has been well-established^1–4^. While the myeloid lineage represents a crucial line of immune defence in the absence of a tumour, tumour-driven distortion of myelopoiesis, resulting in severely altered myeloid phenotypes, is a key mechanism of tumour immune evasion^2,5^. One hallmark of altered myelopoiesis is the skewing of dendritic cell (DC) differentiation to an expansion of myeloid-derived suppressor cells (MDSCs)^6^ and multiple tumour-derived factors have been identified to drive such myeloid deviation^7^. The accumulation of this heterogeneous population of immature myeloid cells and progenitors is strongly associated with tumour progression and unfavourable prognosis across multiple cancer types^8,9^.

DCs are potent antigen-presenting cells (APCs) that are crucial for the orchestration of T cell-mediated tumour elimination. In the tumour microenvironment (TME), however, DCs frequently exhibit a defective phenotype, characterized by markers of immaturity and immunosuppressive activity^10^. Although several pathways have been implicated in DC susceptibility to tumour-derived factors^11–14^, many questions remain open as to which intracellular molecules drive the manifestation of a dysfunctional DC phenotype. This poses considerable limitations to therapeutic strategies aimed at restoring DC functionality in tumours, for example through the delivery of maturation stimuli such as toll-like receptor (TLR) agonists^15,16^.

An intratumoural CD103^+^ DC sub-population has recently been identified to represent a minor, yet the most pivotal APC population mediating anti-tumour immunity in murine models of melanoma^17,18^. High interleukin (IL)-12 secretion^19^, enhanced CD8^+^ cross-priming activity^20^, and the capacity to transport intact tumour antigens to lymph nodes (LN)^21,22^, highlight the importance of CD103^+^ DCs in the priming of an effective cytotoxic anti-tumour T cell response. In melanoma patients, elevated numbers of BDCA3/CD141^hi^ DCs, the equivalent counterpart to murine CD103^+^ DCs in humans, correlate with better prognosis^22–24^. Since the function of certain DC subsets and phenotypes in different cancer types are still not fully resolved, gaining a more thorough understanding of tumour-driven DC plasticity is of urgent interest.

MAPK-activated protein kinase 2 (MK2) is the main downstream target of p38 MAPK^25^. p38-MK2 signalling constitutes a major inflammatory axis with MK2 being responsible for the production of multiple cytokines and chemokines. The pivotal pro-inflammatory function of MK2 in macrophages has been described in various models of systemic inflammation^26,27^. With regard to tumour development, systemic MK2 deletion has been shown to result in reduced skin carcinogenesis^28^ and resistance to inflammation-induced colon carcinoma^29^. Moreover, MK2 acts as cell cycle regulator, coming into play upon p53 mutation^30,31^. In this context, MK2 and several of its downstream effectors have been identified to mediate resistance of tumours to therapy-induced apoptosis^28,32^. Altogether, emerging evidence supports the idea of pharmacological MK2 inhibition as a viable treatment option for both inflammatory and malignant diseases. However, in distinct tissue contexts MK2 has been proposed to mediate negative feedback signalling and dampen on-going inflammatory responses^33–35^. Consistently, we have previously reported a Th1-attenuating function of MK2 in DCs in response to TLR ligation^36^. DC-specific loss of MK2 promotes severe autoimmunity, suggesting a cell type-specific protective role of MK2 in preventing host damage caused by excessive inflammation. However, taking into consideration its diverse functions, whether DC-expressed MK2 modulates anti-tumour immune responses has not been answered to date.

In the present study we therefore set out to address this unresolved question. We used a murine system of CD11c^+^ lineage-specific MK2 deletion (CD11c-Cre *Mapkapk2* ^flox/flox^) and found that MK2 is implicated in immunosuppressive DC activity within the melanoma microenvironment in response to TLR stimulation. Ablation of MK2 in the CD11c^+^ lineage reduced intratumoural DC and MDSC accumulation and promoted the expansion of phenotypically mature CD103^+^ DCs, which favoured the priming of CD8^+^ over CD4^+^ T cells. Along with enhanced infiltration of these T cells into tumours, CD11c-specific loss of MK2 resulted in the generation of a clonally expanded, less exhausted T cell population, altogether reinstating immune control over tumour progression.

## Results

### Tumour growth is reduced in MK2^ΔCD11c^ mice upon activation of endogenous DCs by delivery of tumour antigen and LPS

**Figure 1:**
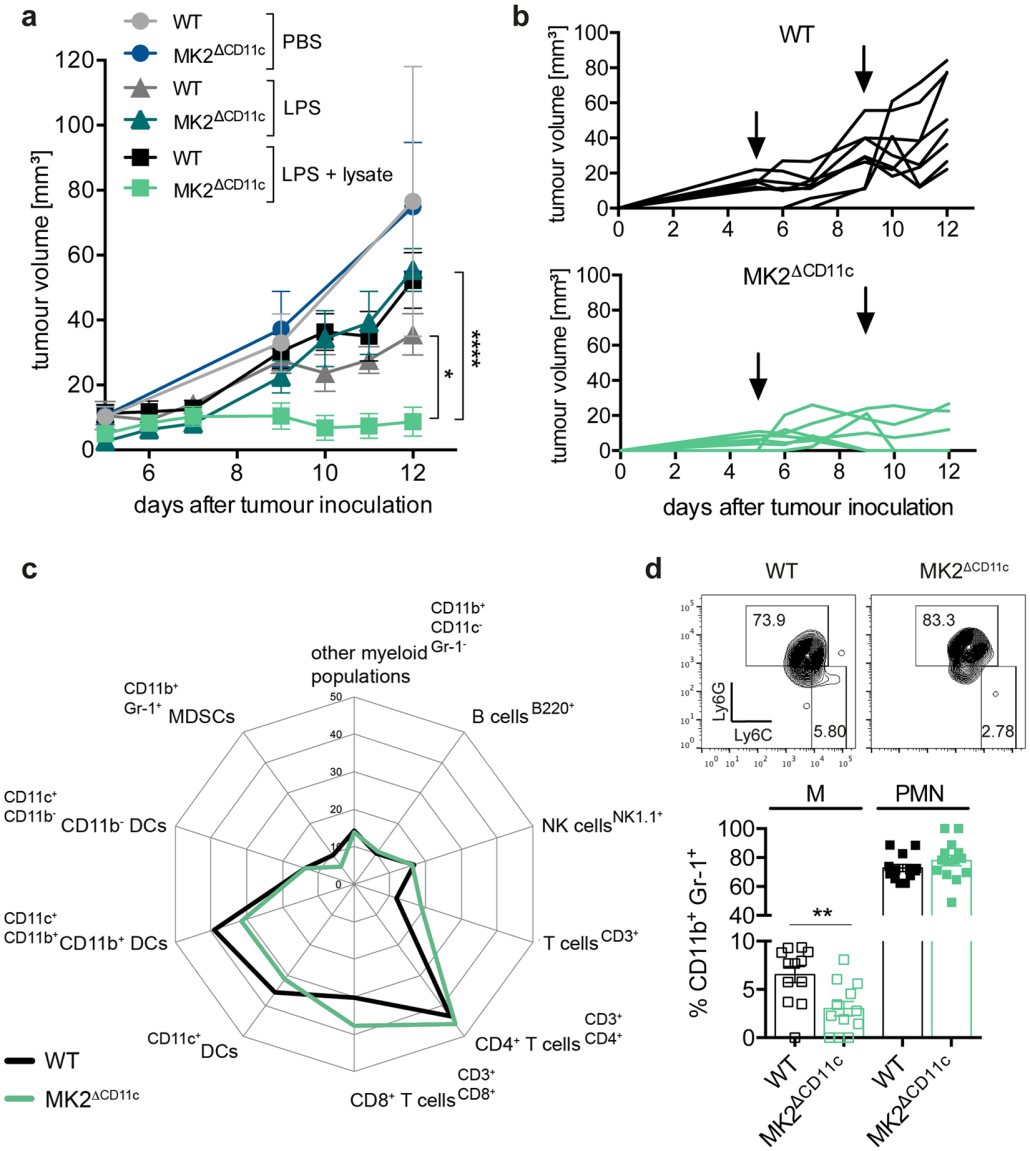
Tumour growth and myeloid cell infiltration is reduced in immunized MK2^ΔCD11c^ mice. (**a**) B16-F10 tumour growth in MK2^ΔCD11c^ and WT mice immunized with PBS, LPS or LPS + B16-F10 lysate on days 5 and 9 post-tumour cell injection. (**b**) Tumour growth curves of individual mice immunized with LPS + B16-F10 lysate. One representative experiment is shown. Arrows indicate days of immunization. (**c**) Spider plot depicting intratumoural immune cell populations in MK2^ΔCD11c^ and WT mice immunized with LPS + B16-F10 lysate as measured by flow cytometry and shown as percentage of live CD45^+^ leukocytes. CD4^+^ and CD8^+^ are percentage of CD3^+^ T cells; CD11b^+^ and CD11b^−^ are percentage of CD11c^+^ MHC-II^+^ DCs. (**d**) Representative plots of Ly6C- and Ly6G-expressing MDSC subsets and cumulative results. Each symbol represents one individual animal. Data are presented as mean ± SEM and in (**a**), (**c**) and (**d**) are pooled from two to three independent experiments (n = 5–8 mice per group each). *P < 0.05, **P < 0.01, ****P < 0.0001. *P*-values were determined using (**a**) repeated-measures two-way ANOVA with Bonferroni correction for multiple comparisons and (**d**) Student’s *t*-test.

We induced melanoma growth in MK2^ΔCD11c^ and wild-type (WT) littermate control mice by subcutaneous injection of the murine melanoma cell line B16-F10, and monitored tumour growth until tumours reached 15 mm in diameter and animals were euthanized. Since we did not detect any difference in either tumour growth (Fig. 1a) or survival (data not shown) between genotypes at baseline, we proceeded to determine whether CD11c^+^ cell-specific loss of MK2 would affect tumour progression upon activation of endogenous DCs via delivery of a danger signal. We therefore administered lipopolysaccharide (LPS) as a potent TLR4 agonist subcutaneously in close proximity to the inguinal (draining) lymph nodes (dLN) on days 5 and 9 post-tumour cell injection. Compared to PBS control groups, tumour growth was delayed in these animals but again no significant differences were observed between genotypes (Fig. 1a and Supplementary Fig. 1a). In addition to delivering an activation trigger, we therefore injected B16-F10 whole cell lysate as tumour antigen together with LPS, to enable DCs to launch an adaptive tumour-directed immune response. Whereas delivery of LPS alone did not affect tumour size in MK2^ΔCD11c^ mice versus WT controls, the combination of TLR agonist and tumour antigen led to a significant reduction and sustained control of tumour growth in MK2^ΔCD11c^ mice and even resulted in tumour clearance in 3 out of 6 animals within 12 days post-tumour cell injection (Fig. 1a,b and Supplementary Fig. 1a). Importantly, no effects were observed using a murine glioblastoma (GL-261) whole cell lysate following the same immunization approach, confirming specificity of tumour-directed DC activation (Supplementary Fig. 1b,c). Critically, the combined B16-F10-directed immunization strategy did not diminish tumour growth in WT littermate controls compared to injection of LPS alone, indicating the importance of MK2 depletion in driving this effect.

The observation of antigen-dependent tumour clearance upon loss of MK2 in the CD11c^+^ lineage led us to investigate how MK2 might contribute to DC-mediated immunosuppression in the TME. We therefore set out to investigate the immune cell composition of the tumour stroma to understand which cellular mechanisms mediate tumour regression in MK2^ΔCD11c^ mice immunized with TLR agonist and tumour antigen.

### Loss of MK2 in the CD11c^+^ lineage alleviates myeloid cell deviation and reduces overall infiltration of melanomas by myeloid cells

**Figure 2:**
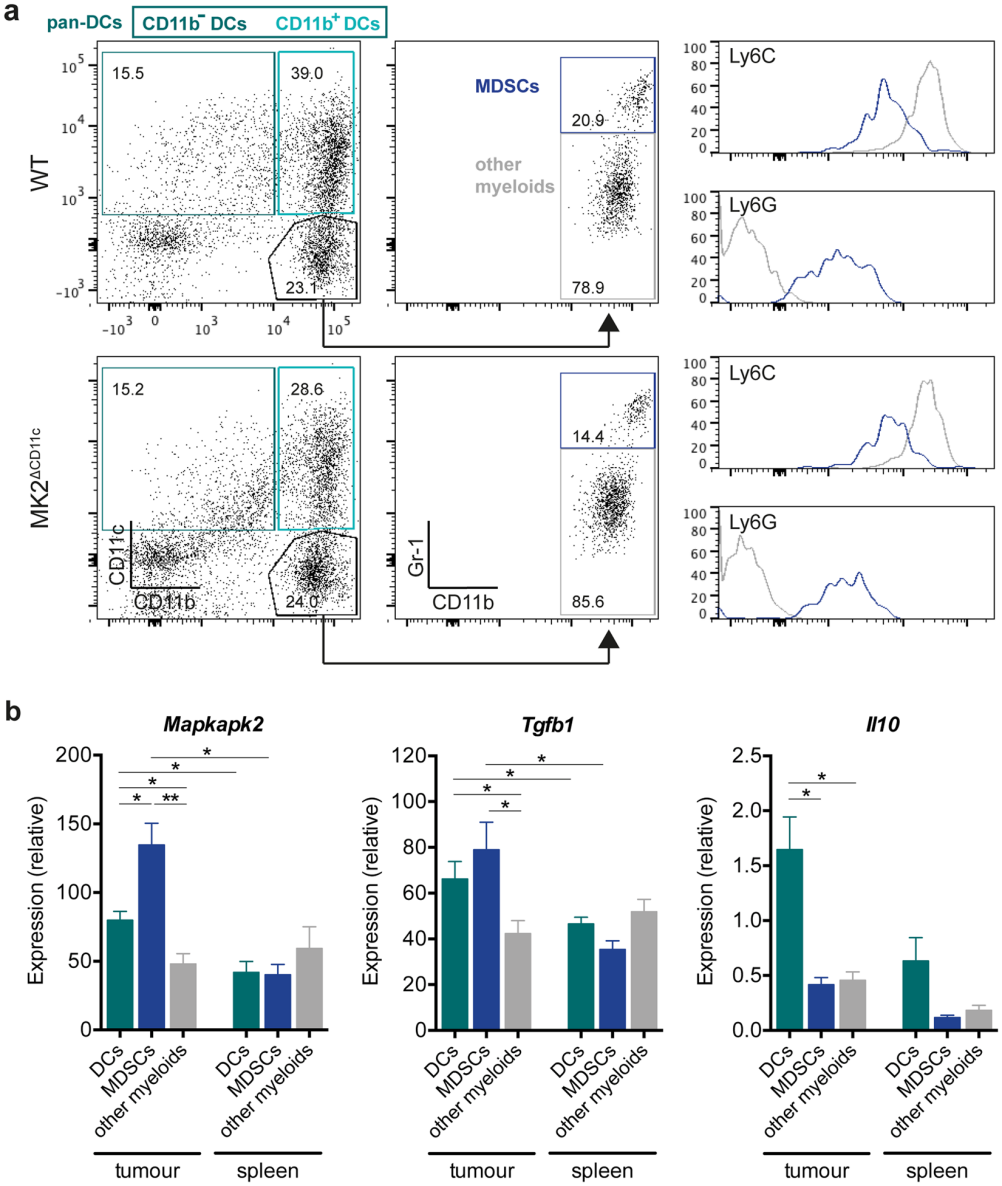
*Mapkapk2* expression correlates with tumour-associated suppressive myeloid cell activity. (**a**) Representative dot plots showing gating strategy of distinct myeloid cell populations. Cells were pre-gated for live, single, CD45^+^ cells. CD11b^+^ and CD11b^−^ DCs were further gated on MHC-II^+^ cells and pan-DCs included both CD11b^+^ and CD11b^−^ DCs. Numbers indicate frequency within parental population. Histograms represent expression levels of Ly6C and Ly6G within MDSCs (blue) and other myeloid cells (grey). (**b**) Gene expression of *Mapkapk2*, *Il10*, and *Tgfb1* in myeloid populations sorted from tumours and spleens of C57BL/6 WT mice as measured by RT-qPCR and normalized to *Ubc* (n = 5). *P < 0.05, **P < 0.01. *P*-values were determined using Mann-Whitney U test.

To examine the immune cell landscape in tumours of MK2^ΔCD11c^ versus WT mice, we isolated tumours on day 12 post-tumour cell injection and analysed the immune infiltrate by flow cytometry. Reflecting the unaffected course of tumour progression between genotypes, PBS and LPS single agent immunization did not induce significant differences in intratumoural leukocyte frequencies (data not shown). However, tumours of MK2^ΔCD11c^ mice showed an overall reduced presence of myeloid cell populations as compared to WT littermate controls when immunized with LPS + tumour cell lysate (Fig. 1c and Supplementary Fig. 1d). Most strikingly, we observed a significant decline in infiltration by highly immunosuppressive immature myeloid CD11b^+^ Gr-1^+^ cells (Fig. 1c and Supplementary Fig. 1d), which have been broadly referred to as MDSCs. Since these cells represent a heterogeneous population of immature myeloid precursors including cells of both granulocytic and monocytic lineages, it is critical to clearly delineate their cellular composition^37^. Across both genotypes, tumour-infiltrating MDSCs comprised mostly cells of polymorphonuclear origin (PMN-MDSC, Ly6G^+^ Ly6C^lo^), which remained unaffected by DC-specific loss of MK2. The monocytic subset (M-MDSC, Ly6G^−^Ly6C^hi^), to which immunosuppressive function, association with poor prognosis and accumulation in advanced melanoma have been ascribed^8,9^, was reduced to 3.0 (±2.6) % in MK2^ΔCD11c^ mice as compared to 6.5 (±2.9) % in WT controls (Fig. 1d). While the presence of other CD11b^+^ myeloid populations (CD11b^+^ CD11c^−^ Gr-1^−^) remained constant (Fig. 1c and Supplementary Fig. 1d), DCs (CD11c^+^ MHC-II^+^) accumulated at significantly lower numbers in tumours of MK2^ΔCD11c^ mice (Fig. 1c and Supplementary Fig. 1d). Notably, the CD11b^+^ CD11c^−^ Gr-1^−^ myeloid population consisted predominantly of cells of monocytic origin, as confirmed by high levels of Ly6C expression (Fig. 2a).

These observations prompted us to investigate whether MK2 is expressed at different levels in these distinct myeloid cell populations. Since myeloid differentiation is severely altered in the context of an evolving TME, we also compared tumour-resident populations to their respective splenic counterparts in tumour-bearing animals. We isolated pan-DCs (CD11c^+^ MHC-II^+^), MDSCs (CD11b^+^ Gr-1^+^) and the remaining CD11b^+^ CD11c^−^ Gr-1^−^ myeloid population from tumours and spleens of melanoma-bearing WT mice at day 12 post-tumour cell injection (Fig. 2a). RT-qPCR analysis revealed elevated expression of *Mapkapk2* in all tumour-resident myeloid subsets compared to the corresponding splenic populations (Fig. 2b). Tumour-resident DCs exhibited pronounced upregulation of *Mapkapk2* by 1.9-fold as compared to splenic DCs and also expressed high levels of *Il10*, *Tgfb1* and *Arg1*, suggesting robust immunosuppressive activity of these cells within the TME (Fig. 2b and Supplementary Fig. 2a). Myeloid populations residing within the spleen showed overall lower levels of all investigated genes, underlining the deviated phenotype and function of myeloid cells within the tumour stroma. Notably, highest *Mapkapk2* expression was observed in MDSCs within the tumour and correlated with elevated levels of prominent immunosuppressive markers *Tgfb1* and *Arg1*. Comparing expression of these markers in intratumoural MDSCs of WT and MK2^ΔCD11c^ mice, however, revealed no significant effect of CD11c-driven MK2 ablation on the MDSC phenotype itself (Supplementary Fig. 2b).

Regarding the differential recruitment of myeloid populations upon CD11c-specific MK2 knock-out, we proceeded to investigate whether loss of MK2 impacts on the production and secretion of specific chemokines in DCs. Since several myelo-attractant chemokines have been described to be regulated via the p38-MK2 pathway in macrophages^38^, we performed gene expression array analysis to determine whether MK2 also controls chemokine regulation in DCs. Examining immature and LPS-matured splenic DCs at 4 and 24 hours post-stimulation, we detected nine chemokines showing significant differences in their expression profile between MK2-proficient and –deficient DCs (Supplementary Fig. 3a). Among these, CCL3 (MIP-1α) and CCL4 (MIP-1β), known to mediate recruitment of myeloid cells^39^, were downregulated upon LPS-induced maturation (Supplementary Fig. 3b,c). We therefore analysed serum levels of these chemokines in tumour-bearing mice immunized with the combination of LPS and whole tumour cell lysate. Whereas CCL3 could only be detected at levels below 0.5 pg/mL in MK2^ΔCD11c^ mice and was undetectable in WT littermate controls, serum CCL4 was slightly decreased from 3.2 (±2.0) to 2.3 (±0.4) pg/mL in MK2^ΔCD11c^ mice (Supplementary Fig. 3d). The fact that multiple cell types other than DCs can secrete these chemokines may provide an explanation as to why only minor differences were observed.

Taken together, upon tumour-directed DC activation, loss of MK2 in CD11c^+^ cells alleviates myeloid deviation leading to reduced MDSC infiltration into the melanoma TME. This is accompanied by a slight reduction in circulating levels of myelo-attractant chemokines such as CCL4. Moreover, MK2 expression is strongly upregulated in suppressive myeloid populations including both tumour-resident MDSCs and DCs and correlates with enhanced expression of known mediators of immunosuppression, such as TGF-β. The significantly decreased presence of DCs themselves in the TME consequently raised the question of how loss of MK2 impacts on the phenotype of tumour-associated DCs.

### CD11c-specific MK2 ablation increases the fraction of CD103-expressing DCs with an enhanced stimulatory phenotype

**Figure 3:**
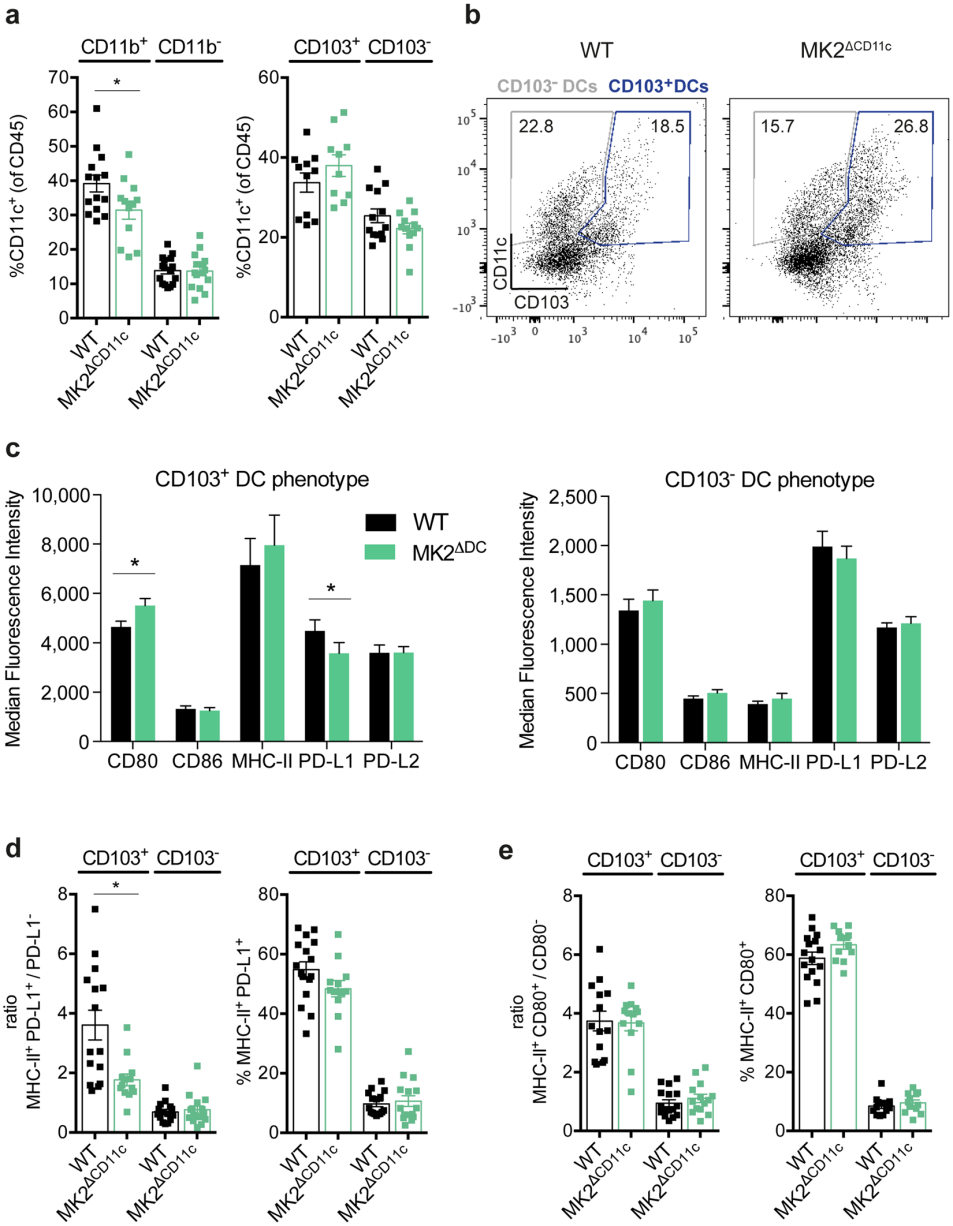
MK2^ΔCD11c^ mice accumulate stimulatory PD-L1^−^ CD103-expressing DCs. (**a**) CD11b and CD103-expressing DCs within tumour-infiltrating CD45^+^ leukocytes as measured by flow cytometry. (**b**) Representative plots showing gating strategy of CD103^+^ and CD103^−^ DCs. Cells were pre-gated for live, single, CD45^+^ CD11c^+^ cells. Frequency within parental population is indicated. (**c**) Surface receptor expression on intratumoural CD103^+^ and CD103^−^ DCs. (**d**) Ratios of PD-L1^+^/PD-L1^−^ cells within MHC-II^+^ DCs and frequency of MHC-II^+^ PD-L1^+^ cells within the indicated parental DC population. (**e**) Ratios of CD80^+^/CD80^−^ cells within MHC-II^+^ DCs and frequency of MHC-II^+^ CD80^+^ cells within the indicated parental DC population. Each symbol represents one individual animal. Data are presented as mean ± SEM and pooled from two to three independent experiments (n = 5–8 mice per group each). *P < 0.05. *P*-values were determined using Student’s *t*-test.

Dissecting the tumour-resident DC (CD11c^+^) compartment more closely with regard to cell surface expression of CD11b and CD103 revealed a robust decline in CD11b^+^ DCs in MK2^ΔCD11c^ mice upon LPS + lysate immunization (Fig. 3a). CD11b^+^ DCs accounted for 39.2% of the whole DC population in WT compared to 31.4% in MK2^ΔCD11c^ tumours, thus forming the most prominent intratumoural DC sub-population. Concurrently, the minor subset of CD11b^−^ DCs prevailed at comparable levels between genotypes. In addition, we detected a fraction of CD103-expressing DCs which was increased upon MK2 knock-out (Fig. 3a,b). As CD103^+^ DCs have been extensively characterized over the last few years to represent a comparatively rare, yet the most potent antigen-presenting and T cell-priming DC population in melanoma^17–19^, we were prompted to analyse this subset further with regard to phenotype and T cell-activating capacity.

Across both genotypes, tumour-resident CD103^+^ DCs expressed 1.5–3-fold higher levels of both co-stimulatory (CD80/B7-1, CD86/B7-2) and –inhibitory (PD-L1/B7-H1, PD-L2/B7-DC) surface molecules as compared to CD103^−^ DCs (Fig. 3c and Supplementary Fig. 4a). Most notably, expression of MHC-II was strongly reduced in CD103^−^ DCs in contrast to CD103^+^ DCs, suggesting their limited antigen-presentation capacity. The absence of MK2 did not significantly alter expression of the aforementioned cell surface molecules within the CD103^−^ sub-population (Fig. 3c). Conversely, in CD103^+^ DCs we observed a two-sided shift towards a more potent immune stimulatory phenotype upon MK2 deletion. While CD80 was upregulated, surface expression of the inhibitory receptor PD-L1 was significantly diminished (Fig. 3c). Consistent with this change in polarization, the distribution of MHC-II/PD-L1 co-expression was altered within this DC subset, with a marked decrease of MHC-II^+^ PD-L1^+^ compared to MHC-II^+^ PD-L1^−^ cells in MK2-deficient CD103^+^ DCs (Fig. 3d and Supplementary Fig. 4b). No comparable effect could be detected in CD103^−^ DCs (Fig. 3d and Supplementary Fig. 4b). Concerning co-stimulation, whereas MHC-II^+^ CD80^+^ DCs were slightly increased within MK2-deficient CD103^+^ DCs, the ratio of MHC-II^+^ CD80^+^/CD80^−^ cells remained unaffected (Fig. 3e and Supplementary Fig. 4c).

To explore whether this combined phenotypical change in CD103^+^ DCs would have an overall impact on the induction of T cells towards recognition and elimination of tumour cells, we investigated the resulting T cell response in MK2^ΔCD11c^ mice upon LPS + tumour antigen immunization.

### DCs accumulate in dLN and yield enhanced CD8^+^ T cell priming upon MK2 deletion

While the presence of tumour-infiltrating stimulatory CD103^+^ DCs indicates T cell engagement directly at the tumour site, regional dLN nevertheless represent the primary site of DC-T cell interaction leading to the activation of tumour-specific T cells. We therefore isolated dLN from MK2^ΔCD11c^ and WT mice immunized with the combination of LPS and tumour antigen at day 12 post-tumour induction and analysed their leukocyte composition by flow cytometry.

**Figure 4:**
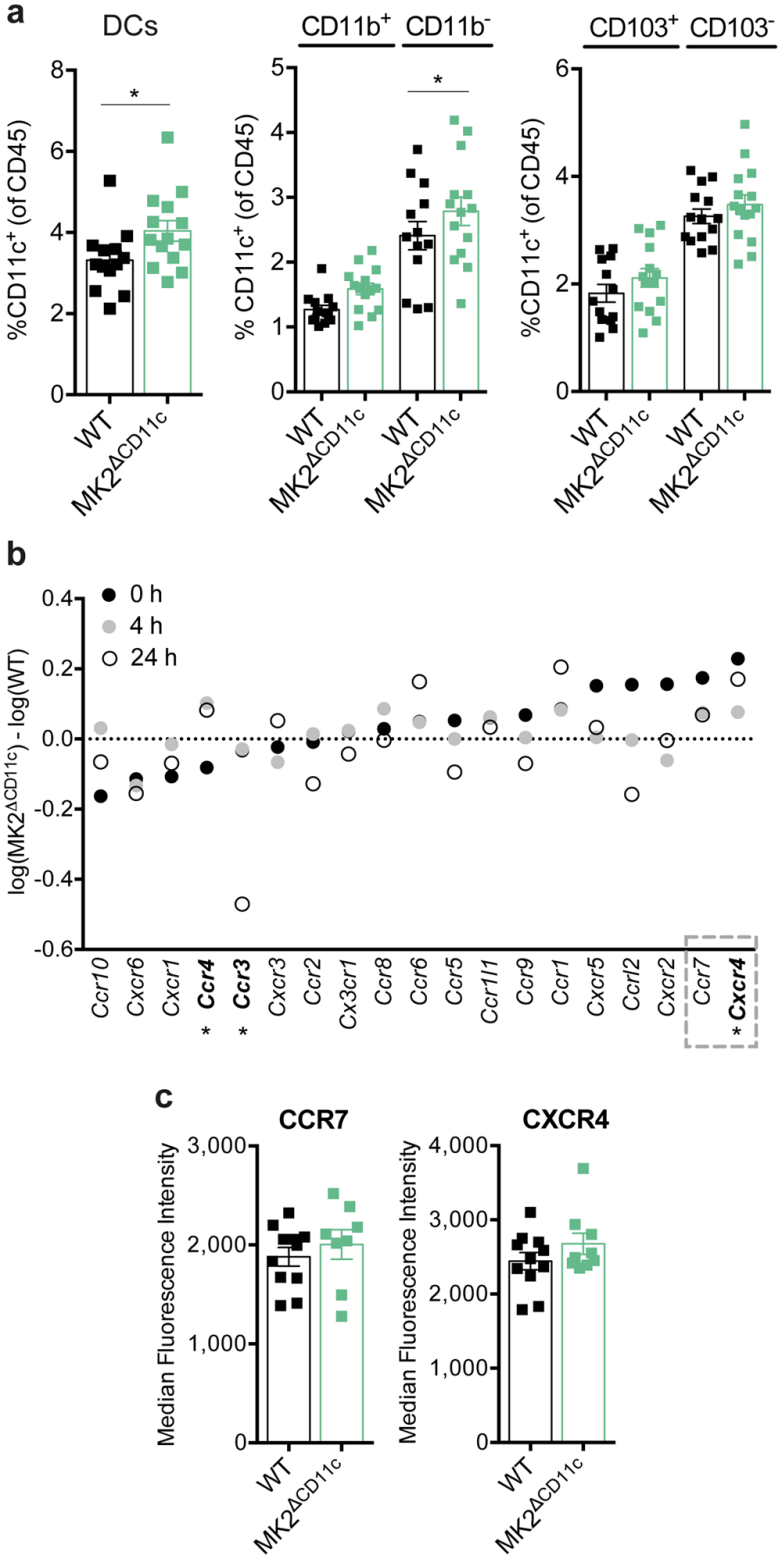
dLN of MK2^ΔCD11c^ mice harbour enhanced numbers of migratory DCs. (**a**) Presence of overall CD11c^+^ MHC-II^+^ DCs, CD11b and CD103-expressing DCs in dLN as measured by flow cytometry. (**b**) Chemokine receptor expression in splenic CD11c^+^ DCs of MK2^ΔCD11c^ and WT mice stimulated for 24 hours with LPS. Differential normalized log2 gene expression between genotypes at 0, 4 and 24 hours post-stimulation is shown (n = 2). Genes printed in bold show significantly different expression levels between genotypes. Grey square marks genes with highest upregulation in MK2^ΔCD11c^ mice at 0 h LPS. (**c**) Surface density (median fluorescence intensity) of CXCR4 and CCR7 on the intratumoural CD11c^+^ DC population analysed by flow cytometry. DCs were gated as live, single, CD45^+^, CD11c^+^ cells. Each symbol represents one individual animal. Data are presented as mean ± SEM and in (**a**) and (**c**) are pooled from two to three independent experiments (n = 5–8 mice per group each). *P < 0.05. *P*-values were determined using Student’s *t*-test.

Although we had previously observed DCs accumulating at lower numbers in MK2^ΔCD11c^ tumours, their presence in dLN was significantly increased in comparison to WT controls (Fig. 4a). In-depth investigation of DC subsets within the overall CD11c^+^ population revealed that this increase occurred primarily in CD11b^−^ DCs (Fig. 4a). Resembling the intratumoural observations, we again found an expansion of the CD103^+^ DC fraction (Fig. 4a). This finding is consistent with recent reports showing that these DCs possess a high migratory potential, enabling them to efficiently transport tumour-antigens to dLN, where effective induction of tumour-reactive T cells takes place^21,22^. Indeed, we observed a slight upregulation of both CCR7 and CXCR4, essential receptors required for LN trafficking of DCs, upon loss of MK2 in tumour-infiltrating DCs (Fig. 4c). We further confirmed the notion that MK2 affects expression of CCR7 and CXCR4 in splenic DCs upon LPS-induced activation, where differential regulation of CXCR4 in particular was strongly pronounced (Fig. 4b and Supplementary Fig. 5a,b).

**Figure 5:**
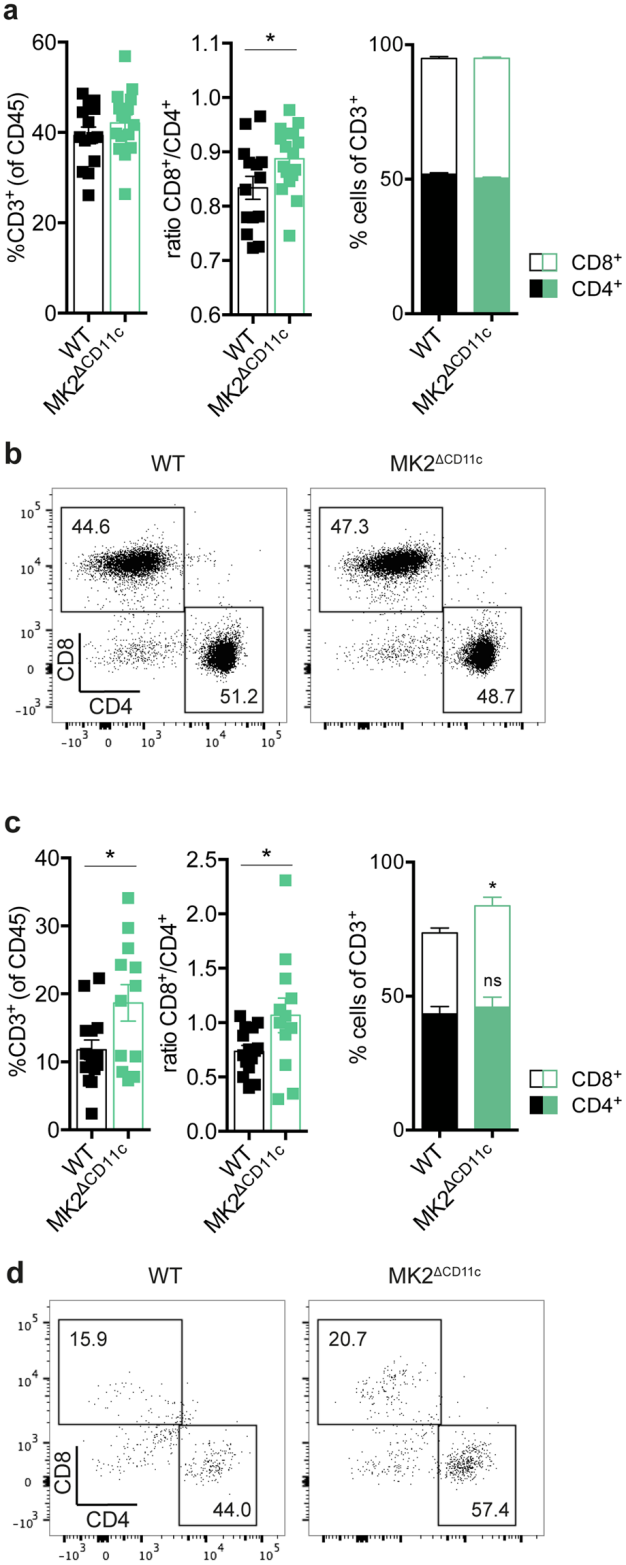
Loss of MK2 increases T cell-stimulatory capacity of DCs. (**a**–**d**) Frequency of CD3^+^ T cells, CD4^+^ and CD8^+^ subsets as well as CD8^+^/CD4^+^ T cell ratio within (**a**,**b**) dLN and (**c**,**d**) tumours as measured by flow cytometry. Bar diagrams showing abundance of CD4^+^ and CD8^+^ T cells within the total CD3^+^ population. (**b**,**d**) Representative dot plots depicting relative frequency of CD4^+^ and CD8^+^ T cell populations within the total CD3^+^ population. Cells were pre-gated for live, single, CD45^+^, CD3^+^ cells. Each symbol represents one individual animal. Data are presented as mean ± SEM and in (**a**) and (**c**) are pooled from two to three independent experiments (n = 5–8 mice per group each). ns, not significant. *P < 0.05. *P*-values were determined using Student’s *t*-test.

As CD103^+^ DCs have been associated with the priming of robust anti-tumour CD8^+^ T cell responses, we analysed the dLN-resident T cell compartment with respect to the presence of CD8^+^ vs. CD4^+^ T cells. Although the overall number of T cells remained similar in MK2^ΔCD11c^ and WT dLN upon LPS + tumour lysate immunization, the ratio of CD8^+^/CD4^+^ cells was significantly altered, favouring the priming of CD8^+^ cytotoxic T lymphocytes (CTLs) upon MK2 knock-out in CD11c^+^ DCs (Fig. 5a,b). Consistently, intratumoural CD8^+^ CTLs increased by a factor of 1.25 from 30.2 (±6.6) % in WT to 37.8 (±11.2) % in MK2^ΔCD11c^ mice (Figs 1c and 5c,d). At the same time, CD4^+^ T cells, which formed the larger fraction of the tumour-infiltrating T cell population, remained unaffected by genotype (Figs 1c and 5c,d).

Thus, the enhanced generation and LN migration of DCs with elevated anti-tumour T cell-priming capacity upon MK2 deficiency gives rise to an overall improved tumour-targeted cytotoxic T cell response, as indicated by robust accumulation of CD8^+^ T cells at the tumour site. To gain deeper insights into the quality of the resulting T cell response, we analysed the intratumoural T cell repertoire in further detail.

### Tumour-directed DC activation results in the generation of a less exhausted, oligoclonally expanded T cell repertoire upon CD11c-specific MK2 deletion

**Figure 6:**
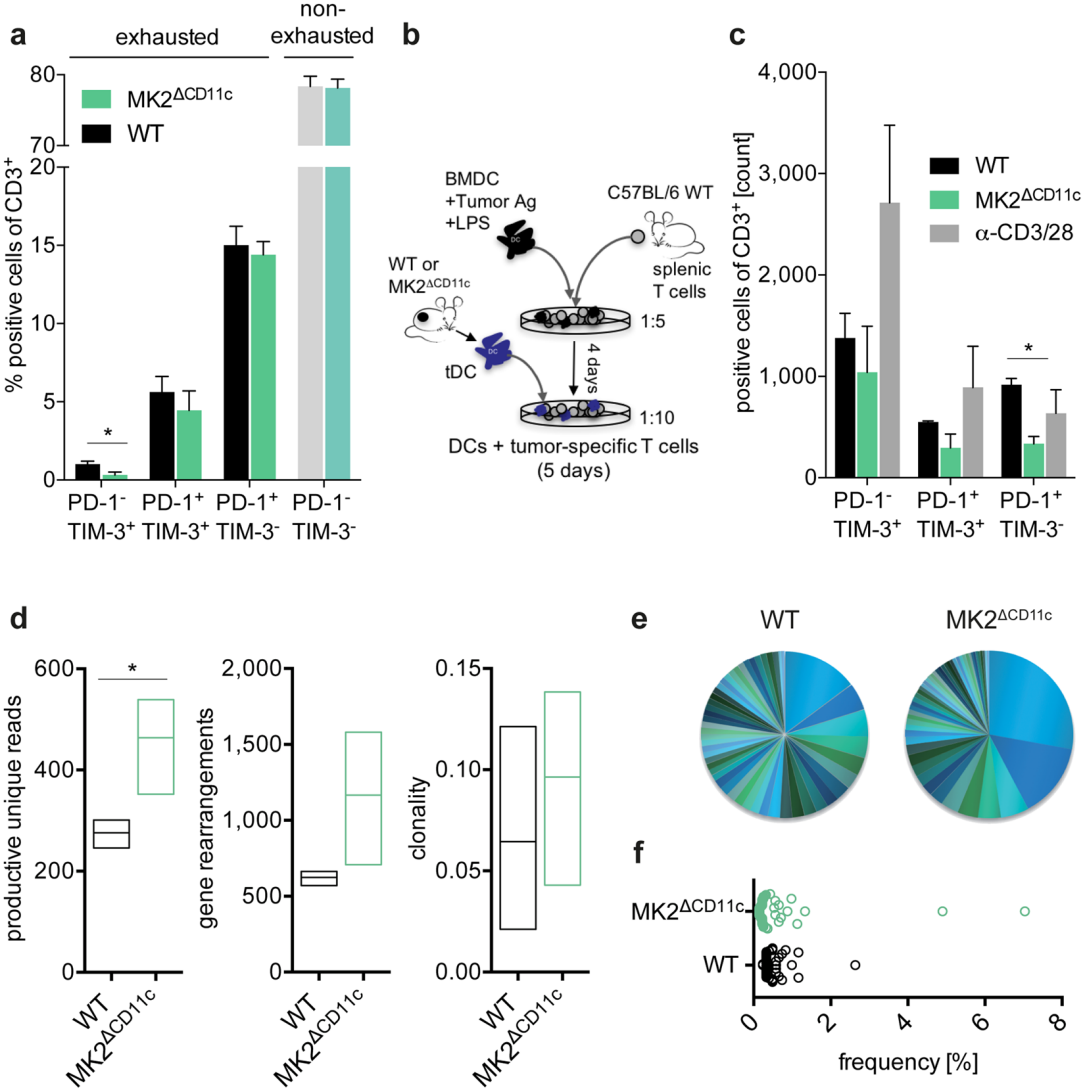
Tumour-infiltrating T cells in MK2^ΔCD11c^ mice appear less exhausted and are oligoclonally expanded. (**a**) PD-1 and TIM-3 expression on tumour-infiltrating T cells as measured by flow cytometry. (**b**) Schematic representation of co-culture experiment analysing PD-1 and TIM-3 expression on splenic T cells pre-expanded by LPS-activated B16-F10 lysate-pulsed BMDCs and co-incubated for 5 days with intratumoural CD11c^+^ DCs. T cells stimulated with αCD3/28 Dynabeads served as activation control. (**c**) Numbers of PD-1 and TIM-3 expressing T cells after co-culture as analysed by flow cytometry (n = 3). (**d**–**f**) Characteristics of intratumoural TCR repertoire. (**d**) Productive unique TCR reads, gene rearrangements and repertoire clonality after normalization (min-max, line indicates mean, n = 6). (**e**) Frequency of 50 highest expressed TCR clones in one representative sample per genotype shown as pie chart and (**f**) cumulative data with each circle representing one individual TCR clone (n = 2). Data are presented as mean ± SEM and in (**a**) are pooled from two to three independent experiments (n = 5–8 mice per group each). *P < 0.05. *P*-values were determined using Student’s *t*-test.

Since the sole accumulation of T cells at the tumour site does not provide information regarding their functionality, we examined their phenotype with respect to the expression of major hallmarks of T cell exhaustion and dysfunction, PD-1 and TIM-3^40^. Whereas the majority of the intratumoural T cell pool in LPS + tumour antigen-immunized mice did not express either receptor, we nevertheless observed a significant decline in PD-1^−^ TIM-3^+^ cells and an overall trend towards reduced inhibitory receptor expression upon CD11c^+^ lineage-specific MK2 knock-out (Fig. 6a). We further confirmed this less exhausted phenotype in an *in vitro* T cell activation assay by co-culturing spleen-derived T cells with antigen-pulsed LPS-activated DCs isolated from tumours of MK2^ΔCD11c^ and WT littermate control mice (Fig. 6b,c and Supplementary Fig. 6a). Of note, tumour-resident MK2^ΔCD11c^ DCs led to the differentiation of almost 50% fewer PD-1-expressing T cells *in vitro* (Fig. 6c and Supplementary Fig. 6a). Regarding T cell activation and expansion, DC-targeted MK2 ablation did not affect either proliferation or CD25 upregulation on T cells and tumour-resident DCs of both genotypes failed to induce an overall T cell expansion *in vitro* (Supplementary Fig. 6b,c).

**Figure 7:**
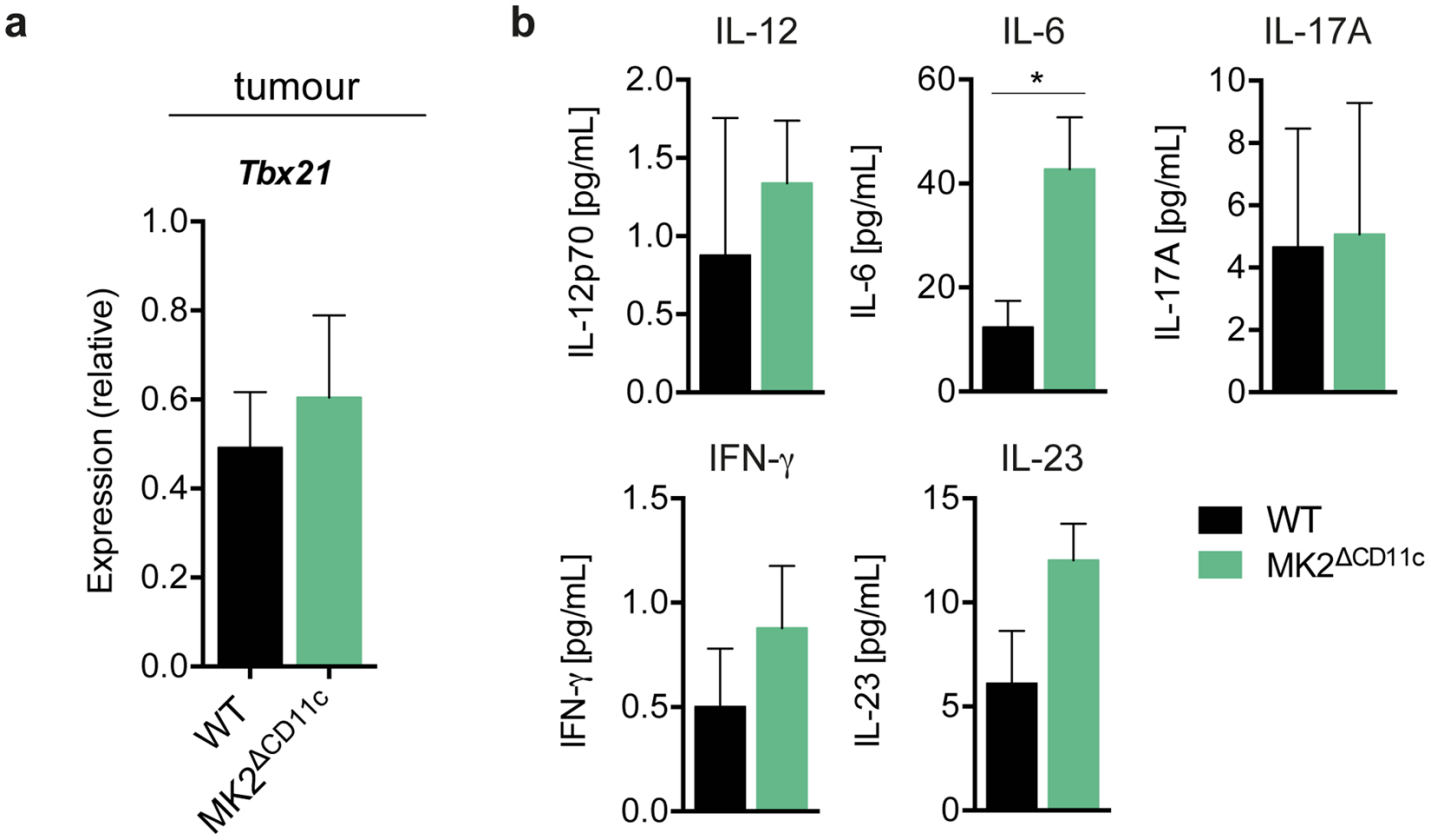
CD11c-specific MK2 deletion supports Th1 polarization. (**a**) *Tbx21* expression in frozen tumour tissue as measured by by RT-qPCR and normalized to *Ubc*. (**b**) Serum IL-12p70, IFN-γ, IL-6, IL-23, and IL-17A in tumour-bearing mice as analysed by ProcartaPlex™ Multiplex Immunoassay (n = 5). Data are presented as mean ± SEM and in (**a**) are pooled from two to three independent experiments (n = 5–8 mice per group each). *P < 0.05. *P*-values were determined using (**a**) Mann-Whitney U test and (**b**) Student’s *t*-test.

Dissecting the tumour-infiltrating T cell receptor (TCR) repertoire of MK2^ΔCD11c^ versus WT mice revealed a robust increase in productive unique TCRs and numbers of gene re-arrangements along with enhanced clonality of the overall TCR repertoire upon LPS + lysate immunization (Fig. 6d–f). This observation implies a more effective generation and expansion of tumour-targeted T cell clones, corroborating the elevated accumulation of CTLs in tumours of MK2^ΔCD11c^ mice. Notably, we didn’t detect single dominant TCR clones arising in large numbers, but rather an overall increase in oligoclonality, which we attribute to the whole-lysate immunization strategy (Supplementary Table 1). Furthermore, this was accompanied by a trend towards increased expression of *Tbx21* in tumour and *Ifng* in both tumour and spleen tissue of MK2^ΔCD11c^ mice (Fig. 7a and Supplementary Fig. 6d), which suggests a more Th1-polarized T cell response upon deletion of MK2 in DCs that is consistent with our previous observations in the context of systemic inflammation and autoimmunity^36^. Serum levels of IL-12 and IFN-γ were also slightly increased in immunized MK2^ΔCD11c^ mice on day 12 post-tumour cell injection (Fig. 7b). Additionally, we detected a pronounced increase in IL-6 and IL-23 (Fig. 7b), representing critical mediators of inflammation. Simultaneously, IL-17A remained unaffected (Fig. 7b), indicating no enhanced Th17-polarized response.

In conclusion, loss of MK2 in the DC lineage results in the induction of a potent tumour-targeted cytotoxic T cell response upon local simultaneous delivery of a TLR agonist and tumour antigen. Besides increased priming and accumulation of CD8^+^ T cells at the tumour site, tumour-infiltrating effector T cells exhibit a less exhausted phenotype with reduced expression of immune checkpoint receptors PD-1 and TIM-3. Finally, high clonality of the intratumoural TCR pool indicates effective expansion of tumour-reactive T cell clones, together enabling sustained tumour growth control.

## Discussion

Tumour cells develop intricate mechanisms to evade recognition by the immune system. The highly versatile nature of DCs makes them prone to manipulation by tumour factors. As a result, tumour-associated DCs often display a dysfunctional phenotype and contribute to tumour immune escape^2,10^. We identified the p38 downstream effector kinase MK2 as an intracellular mediator of tumour-driven DC malfunction in the presence of TLR-activating signals and tumour antigen. This has important implications with regard to therapeutic strategies aiming to maintain or restore DC maturity. The administration of adjuvant DC-activating danger signals, including TLR agonists, is currently being harnessed to enhance responses to different treatment regimens^15,16^. However, limited success has been achieved so far. One explanation is provided by the observation that following transient activation, a majority of DCs revert to a state of malfunction, presumably due to complex interactions within intracellular signalling circuits. Even though many factors in the TME have been identified to induce DC dysfunction and modulate their response to TLR ligation^12,13,41^, the intracellular mediators translating these signals into a regulatory phenotype are unknown. Our finding herein of significantly diminished tumour growth in MK2^ΔCD11c^ mice in response to TLR activation and delivery of tumour antigen highlights MK2 as one potential candidate.

Tumours are capable of exploiting immune regulatory mechanisms that are required to maintain peripheral tolerance in a healthy organism, as exemplified by prominent immune checkpoint molecules^40^. Our previous studies revealed MK2 as a molecular switch mediating a shift from inflammatory to immunosuppressive DC activity upon prolonged TLR stimulation, which proved to be crucial for the prevention of autoimmune reactions emerging from an excessive inflammatory response^36^. Consistently, others have reported similar patterns of MK2 involvement in negative feedback signalling in other cell types^33,35^. These observations are contrasted by studies revealing that systemic loss of MK2 causes enhanced susceptibility to infections^27^ and reduces inflammation-induced tumour growth^28,29^. Collectively, these aspects underline the importance of MK2 as a central mediator of inflammation, with a crucial feedback function in DCs that is required for the tight control and timely resolution of inflammatory processes. This function, however, is essentially linked to simultaneous antigen stimulation. The significance of such self-limiting features of negative regulation have recently been highlighted with reference to other key inflammatory pathways^42^. Consequently, our findings suggest that MK2 represents an intracellular immune checkpoint in DCs. While MK2 is required for DC-mediated immune homeostasis, tumour cells seem to hijack this function to arrest DCs in a dysfunctional state. Several tumour-derived molecules act as major drivers of DC malfunction^13,43^ and engage the p38-MK2 pathway for signal transduction^44,45^, exemplifying how MK2 could be exploited to relay environmental cues to modulate DC behaviour. Based on these observations, we propose that MK2 signalling conveys tumour-driven DC suppression and restricts DC activation by TLR agonists in the tumour stroma.

While abundance and phenotype of DC populations vary greatly across cancer types^1,11^, one specific DC subset has recently emerged as the pivotal APC population mediating rejection of melanoma cells. Designated as DC2 by Broz *et al*., these cells are CD103^+^ and exhibit a signature of enhanced stimulatory activity^17,22^. We show here that CD103-expressing DCs are enriched among the overall MK2-deficient DC population. In accordance with their high T cell-engaging capacity, we detected a robust increase in MHC-II concomitant with alterations in co-stimulatory and inhibitory molecules on the surface of these APCs as compared to their CD103^−^ counterparts, which was amplified upon MK2 ablation. Most strikingly, PD-L1 expression was significantly diminished, and the ratio of MHC-II^+^ PD-L1^+^ double positive cells over PD-L1^−^ cells was tilted in favour of PD-L1^−^ APCs upon loss of MK2. Considering the importance of antigen presentation for successful priming of tumour-reactive CTLs, this shift indicates improved antigen-specific T cell activation in the absence of MK2. The implications of these observations are particularly interesting as PD-L1 has been suggested as the dominant pathway of immunosuppression by tumour-associated DCs^15^. Moreover, phenotypically mature CD103^+^ DCs have been reported to be indispensable for response to immune checkpoint therapy targeting PD-L1 in melanoma^21^. Our findings here delineate the importance of MK2 in the regulation of stimulatory and inhibitory surface receptors on such DCs and suggest MK2 being responsible for a limited expansion of stimulatory DCs within the intratumoural DC population.

Corresponding to their enhanced stimulatory signature, CD103-expressing DCs are crucial for the transport of intact tumour antigens to regional dLNs^21^. We found that MK2 deletion resulted in differential regulation of a number of chemokine receptors on DCs, with CCR7 and CXCR4 representing the most highly upregulated examples. While CCR7 represents a general key regulator for DC LN migration, CXCR4 is required for the movement of cutaneous DCs and epidermal Langerhans cells^46,47^. In our melanoma model, elevated expression of CXCR4 upon MK2 deletion correlated with an accumulation of DCs in dLNs. Furthermore, the proportion of stimulatory CD103-expressing DCs was increased in lymphoid tissues. Taken together, this suggests improved trafficking of tumour-associated DCs bearing a mature, stimulatory signature to regional LN in the absence of MK2.

Another hallmark of melanoma-associated CD103^+^ DCs is an elevated CD8^+^ cross-priming activity, highlighting their significance in directing a cytotoxic anti-tumour T cell response^20^. Accompanying elevated numbers of CD103^+^ DCs upon MK2 deletion, we observed a shift in both intratumoural and LN-resident T cell populations towards a higher presence of CD8^+^ T cells upon immunization with LPS + tumour antigen. Regarding T cell functionality, we previously reported enhanced proliferation and cytotoxicity of CD8^+^ T cells following priming by MK2-deficient DCs in the context of inflammation^36^. The observation here that ablation of MK2 in the CD11c^+^ lineage yielded enhanced accumulation of CD8^+^ T cells at the tumour, which exhibited a clonally enriched TCR repertoire, suggests successful expansion of tumour-directed T cell clones. Even more importantly, MK2-deficient DCs induced lower levels of inhibitory receptors PD-1 and TIM-3 on both CD4^+^ and CD8^+^ T cells. Together, the sum of these changes in the T cell response appears sufficient to enable tumour-reactive T cells to regain control over tumour progression.

Besides an impaired T cell response, a heterogeneous population of CD11b^+^ Gr-1^+^ immature myeloid cells or MDSCs represents one of the most prominent immunosuppressive mediators that accelerates tumour immune evasion. Loss of MK2 in the CD11c^+^ lineage reduced overall myeloid tumour infiltration and led to diminished numbers of intratumoural MDSCs. Low serum levels of myelo-attracting chemokines CCL3 and CCL4 furthermore suggest an involvement of MK2 in DC-mediated recruitment of suppressive myeloid cells into the TME. Moreover, release of CCL4 has been linked to recruitment of regulatory T cells^48^ and tumour-promoting Th17 cells^49^. Nevertheless, we cannot exclude the possibility that MK2 impacts directly on tumour-driven distortion of myeloid differentiation, making DCs susceptible to assume an MDSC-like phenotype. Supporting this hypothesis, we found MK2 expression to be elevated in MDSCs as compared to other myeloid cell types. This increase correlated with markers of immunosuppression TGF-β, Arg-1 and IL-10^37^. Consistently, our prior studies revealed IL-10 secretion from DCs to be stringently regulated by MK2 upon TLR-induced inflammation^36^. However, CD11c-driven MK2 ablation did not affect the phenotype of MDSCs itself, hence suggesting a dominant effect on the mobilization and recruitment of suppressive myeloid cells to the TME.

**Figure 8:**
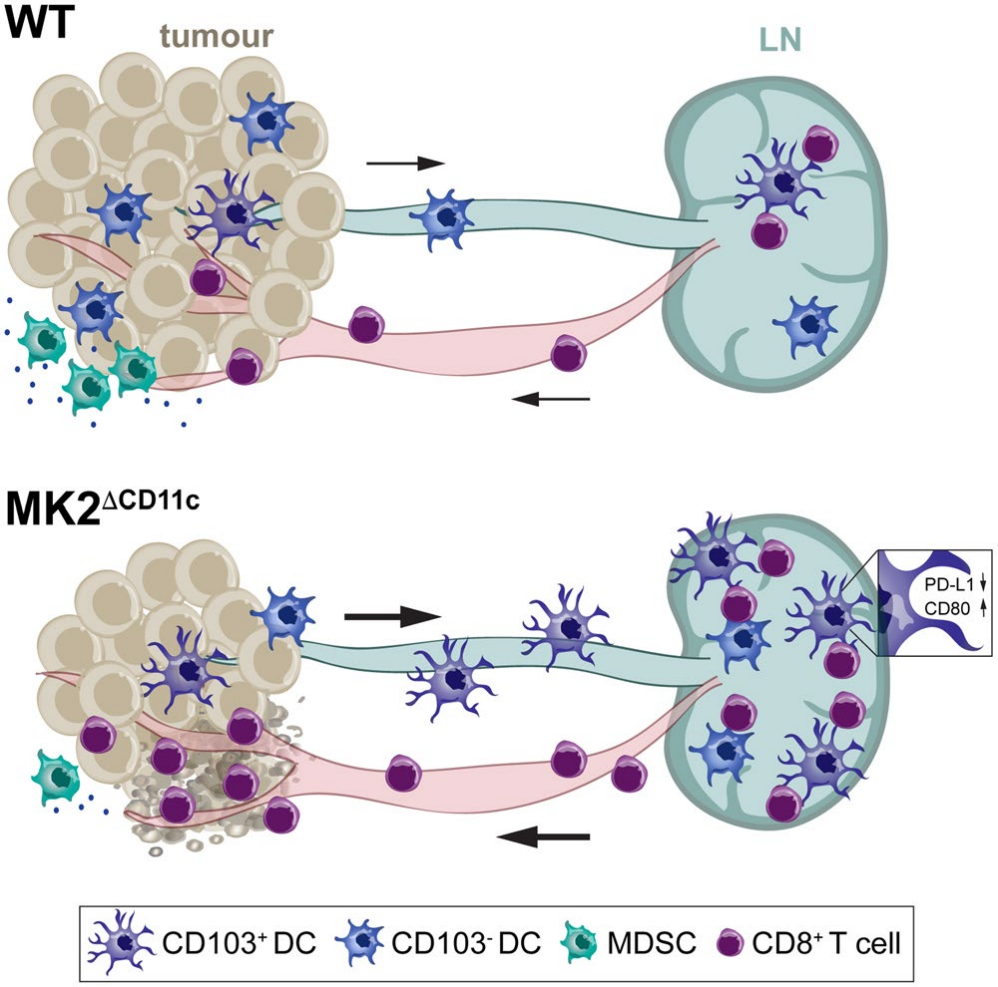
Schematic model of DC-orchestrated anti-tumour immune response upon MK2 deletion. CD11c-specific MK2 deletion enables DCs to orchestrate a potent anti-tumour T cell response upon delivery of a TLR-activating agonist in combination with tumour antigen. In addition to the expansion of immune-stimulatory CD103-expressing DCs, MK2 deletion results in enhanced dLN accumulation of DCs yielding an effective anti-tumour CD8^+^ T cell response. CD8^+^ CTLs subsequently infiltrate into tumours and mediate stable elimination of tumour cells. Additionally, CD11c-specific MK2 ablation leads to a profound decrease of suppressive myeloid populations, such as MDSCs, at the tumour site, further reverting an immunosuppressive milieu within the tumour microenvironment.

In summary, we show that MK2 expression in tumour-associated DCs contributes to tumour immune evasion on three levels (Fig. 8): (i) hindering the sustained acquisition of a mature and functional DC phenotype, (ii) dampening an anti-tumour CD8^+^ T cell response, and (iii) mediating expansion of suppressive myeloid populations at the tumour site. In tumour cells, the identification of MK2 as an alternative cell cycle checkpoint, mediating resistance to apoptosis upon p53 mutation, has led to the emergence of MK2 as a promising target for combinatorial cancer therapies^26^. When envisioning administration of pharmacological MK2 inhibitors, it will therefore be crucial to dissect concurrent effects on immune cells both within tumour stroma and periphery. Our data suggest a synergistic effect of MK2 inhibition on DC-orchestrated anti-tumour immune responses. The combination of MK2 inhibitors with conventional anti-cancer therapies on the one hand, and immune modulatory strategies aiming at DC re-activation on the other, holds considerable promise. Future preclinical studies exploring different therapy combinations will thus be of critical importance to evaluate the potential of targeting MK2 for the treatment of cancer.

## Methods

### Mice

CD11c-Cre *Mapkapk2* ^flox/flox^ (MK2^ΔCD11c^) mice were generated and genotyped as previously described^36^. C57BL/6N wild-type mice were purchased from the Division for Laboratory Animal Science and Genetics of the Medical University of Vienna (Himberg/Vienna, Austria). For all experiments, female and male mice at 8–14 weeks of age were used. All animal experiments were performed in accordance with the institutional guidelines and approved by the Animal Care and Use Committee of the Medical University of Vienna (GZ: 856861/2013/16).

### Melanoma model

The B16-F10 murine melanoma cell line was purchased from the ATCC (CRL-6475) and maintained in DMEM + GlutaMAX™ (Gibco) supplemented with 10% FCS (Sigma-Aldrich), 100 U/mL penicillin, and 100 μg/mL streptomycin (Gibco). 3–4 passages post-thawing, cells were tested for mycoplasma (MycoAlert™, Lonza) and subsequently 10^5^ B16-F10 cells injected subcutaneously into left and right lateral flanks of mice. Five days post-tumour cell injection mice were immunized with either PBS, LPS (*E*.*coli* O111:B4, Calbiochem, 1 mg/kg) alone or in combination with either B16-F10 or GL-261 (cell line kindly provided by J. Hlavaty, University of Veterinary Medicine Vienna) whole cell lysate (0.4 mg/kg) subcutaneously proximal to the inguinal lymph nodes. Immunization was repeated four days later. Cell lysates were prepared by four intervening freeze/thaw cycles. Tumour volume was measured using a digital calliper and calculated as V = (width^2^ × length)/2, with length representing the largest and width representing the perpendicular diameter.

### Flow cytometry and sorting of myeloid populations

On day 12 post tumour cell injection, animals were euthanized and organs isolated. Single cell suspensions of whole spleen and tumour tissue were obtained by enzymatic digestion using 10 mg/mL collagenase D and 20 U/mL DNase I (both Roche), followed by red blood cell lysis. Tumour-draining (inguinal) LN were meshed and filtered at 70 μm. Cells were stained with the following antibodies: CD45.2-APC-eFluor780 (104), CD11b-APC (M1/70), CD11c-eFluor450 (N418), CD3ε-FITC (145-2C11), CD4-PerCP-eFluor710 (RM4-5), CD4-PerCP-Cy5.5 (RM4-5), CD4-eVolve605 (RM4-5), CD8a-PE (53–6.7), CD45R (B220)-Alexa Fluor 700 (RA3-6B2), NK1.1-PE-Cy7 (PK136), Gr-1-FITC (RB6-8C5), CD274 (PD-L1)-PE-Cy7 (MIH5), CD273 (PD-L2)-PerCP-eFluor710 (122), MHC-II I-Ab-FITC (AF6-120.1), MHC-II I-Ab-PerCP-eFluor710 (AF6-120.1), CD279 (PD-1)-PE-Cy7 (J43), CD279 (PD-1)-APC-eFluor780 (J43), TIM-3-PE (8B.2C12), CD197 (CCR7)-Alexa Fluor 700 (4B12), CD184 (CXCR4)-PerCP-eFluor710 (2B11) (all eBioscience); CD8a-Brilliant Violet 510 (53-6.7), F4/80-PerCP (BM8), CD86-Brilliant Violet 650 (GL-1), CD103-Brilliant Violet 510 (2E7) (all BioLegend); CD3ε-PE-CF594 (145-2C11), Ly6C-Brilliant Violet 605 (AL21), Ly6G-PE (1A8), CD80-PE-CF594 (16-10A1) (all BD Biosciences). Dead cells were stained using 7-AAD (eBioscience). Flow cytometry was performed on a BD LSRFortessa™ flow cytometer and data analysed using FlowJo 10.0.5 (Treestar). Live DCs (CD45.2^+^ 7-AAD^−^ CD11c^+^ MHC-II^hi^), MDSCs (CD45.2^+^ 7-AAD^−^ CD11c^−^ CD11b^+^ Gr-1^hi^) and other myeloid cells (CD45.2^+^ 7-AAD^−^ CD11c^−^ CD11b^+^ Gr-1^−^) were sorted from whole spleen and tumour single cell suspensions on a BD FACSAria™ II flow cytometer.

### RT-qPCR

RNA was isolated from fresh sorted myeloid cell populations or frozen splenocyte single cell suspensions using the RNeasy Mini Kit (Qiagen) and reverse-transcribed using the High-Capacity cDNA Reverse Transcription Kit (Thermo Fisher). Quality control of RNA isolated from frozen samples was done using the Experion™ RNA StdSens Assay (Bio-Rad). TaqMan probes (Life Technologies) were used to quantify expression of *Mapkapk2* (Mm01288465_m1), *Tgfb1* (Mm01178820_m1), *Il10* (Mm01288386_m1), *Cxcr4* (Mm01996749_s1), *Ccr7* (Mm99999130_s1), *Ccl3* (Mm00441259_g1), *Ccl4* (Mm00443111_m1), *Tbx21* (Mm00450960_m1) and *Ifng* (Mm01168134_m1) normalized to *Ubc* (Mm02525934_g1) as a housekeeping control.

### *In vitro* T cell activation assay

For assessment of T cell activation, bone marrow-derived dendritic cells (BMDCs) were generated as described previously^50^ and pulsed for four hours with 10 μg/mL B16-F10 freeze/thaw lysate in the presence of 100 ng/mL LPS (*E*. *coli* O111:B4, Calbiochem). BMDCs were then cultured with CD3^+^ T cells isolated from spleens of C57BL/6N wild-type mice (Pan T cell Isolation Kit II, Miltenyi Biotec) for four days at a 1:5 ratio to generate tumour-specific T cells. After four days, T cells were harvested. DCs were sorted from tumour (tDCs) single cell suspensions on day 12 post-tumour cell injection following dead cell removal (Miltenyi Biotec) on a BD FACSAria™ II flow cytometer. tDCs were then co-incubated with tumour-specific T cells at a 1:10 ratio in 96-well U-bottom plates and cultures analysed by flow cytometry five days later. T cells stimulated with 1 μL αCD3/CD28 Dynabeads (Life Technologies)/100,000 cells served as activation control.

### Serum cytokine/chemokine analysis

Murine sera collected on day 12 post-tumour cell injection were analysed using the ProcartaPlex™ Multiplex Immunoassay technology (Affymetrix) combining simplex kits to detect IFN-γ, IL-6, IL-12p70, IL-17A, IL-23, MIP-1α (CCL3) and MIP-1β (CCL4) according to the manufacturer’s instructions.

### Splenic DC gene expression analysis

CD11c^+^ cells were purified from whole spleen single cell suspensions of MK2^ΔCD11c^ and WT littermate control mice by magnetic bead isolation (Miltenyi Biotec) according to the manufacturer’s instructions. Cells were stimulated with 100 ng/mL LPS (*E*.*coli* O111:B4, Calbiochem) for 4–24 hours. RNA was isolated and submitted for quality control (Agilent Bioanalyzer RNA 6000 Nano), labelling and measurement on a Mouse Gene 2.0 ST Array (Affymetrix) to the Core Facility Genomics at the Medical University of Vienna (kindly performed by Markus Jeitler). Data were normalized by RMA Sketch (Expression Console, Affymetrix) and analysed using one-way between-subject ANOVA (unpaired) with a p value < 0.05 designating statistical significance.

### TCR sequencing

DNA was isolated from formalin-fixed paraffin-embedded B16-F10 tumours using the AllPrep DNA/RNA FFPE Kit (Qiagen) and submitted for amplification and survey level sequencing of TCRB CDR3 via the immunoSEQ platform (Adaptive Biotechnologies). Data were normalized and analysed using the immunoSEQ analyzer software toolset.

### Statistical analysis

All animal experiments included 5–8 mice per group and were repeated independently at least twice. Tumour size was measured in a blinded manner with regard to experimental groups. Randomization of mice to groups was done based on tumour size on day 5 post-tumour cell inoculation. Data are presented throughout as mean ± SEM with each symbol representing one individual animal and were analysed by two-tailed unpaired or paired Student’s *t*-test. RT-qPCR data were analysed by Mann-Whitney U test. Tumour growth curves were analysed by repeated-measures two-way ANOVA and Bonferroni correction for multiple comparisons. Significance levels are indicated as ns (not significant), *P < 0.05, **P < 0.01; ***P < 0.001 and ****P < 0.0001. All statistical analyses were done using GraphPad Prism 6.0.

### Data availability

The microarray datasets are available in the GEO repository (accession number: GEO:GSE96628). All other datasets generated during the current study are available from the corresponding authors upon reasonable request.

## Electronic supplementary material

Supplementary information
