# Supplementary material for: Loss of MAPK-activated protein kinase 2 enables potent dendritic cell-driven anti-tumour T cell response
Source: Sci Rep. 2017 Sep 18;7:11746. doi: 10.1038/s41598-017-12208-7 (PMC5603533; doi:10.1038/s41598-017-12208-7)
Supplement: Supplementary file 1 — Supplementary information [file 41598_2017_12208_MOESM1_ESM.pdf]

SUPPLEMENTARY INFORMATION

Loss of MAPK-activated protein kinase 2 enables potent dendritic cell-driven anti-tumour T cell response

Klara Soukup[1,4*], Angela Halfmann[1], Barbara Dillinger[1], Fiona Poyer[1], Katharina Martin[1], Bernadette Blauensteiner[1], Maximilian Kauer[2], Mario Kuttke[3], Gernot Schabbauer[3] and Alexander M. Dohnal[1,5*]

[1]Tumour Immunology, St. Anna Kinderkrebsforschung, Children's Cancer Research Institute, Vienna, Austria

[2]Bioinformatics, St. Anna Kinderkrebsforschung, Children's Cancer Research Institute, Vienna, Austria

[3]Institute for Physiology, Centre for Physiology and Pharmacology, Medical University of Vienna, Vienna, Austria

[4]Current address: Department of Fundamental Oncology, Ludwig Institute for Cancer Research, University of Lausanne, Lausanne, Switzerland

[5]Current address: APEIRON Biologics AG, Vienna, Austria

[*]Correspondence and requests for materials should be addressed to K.S. (email: klara.soukup@unil.ch) or A.M.D. (email: alexander.dohnal@apeiron-biologics.com)

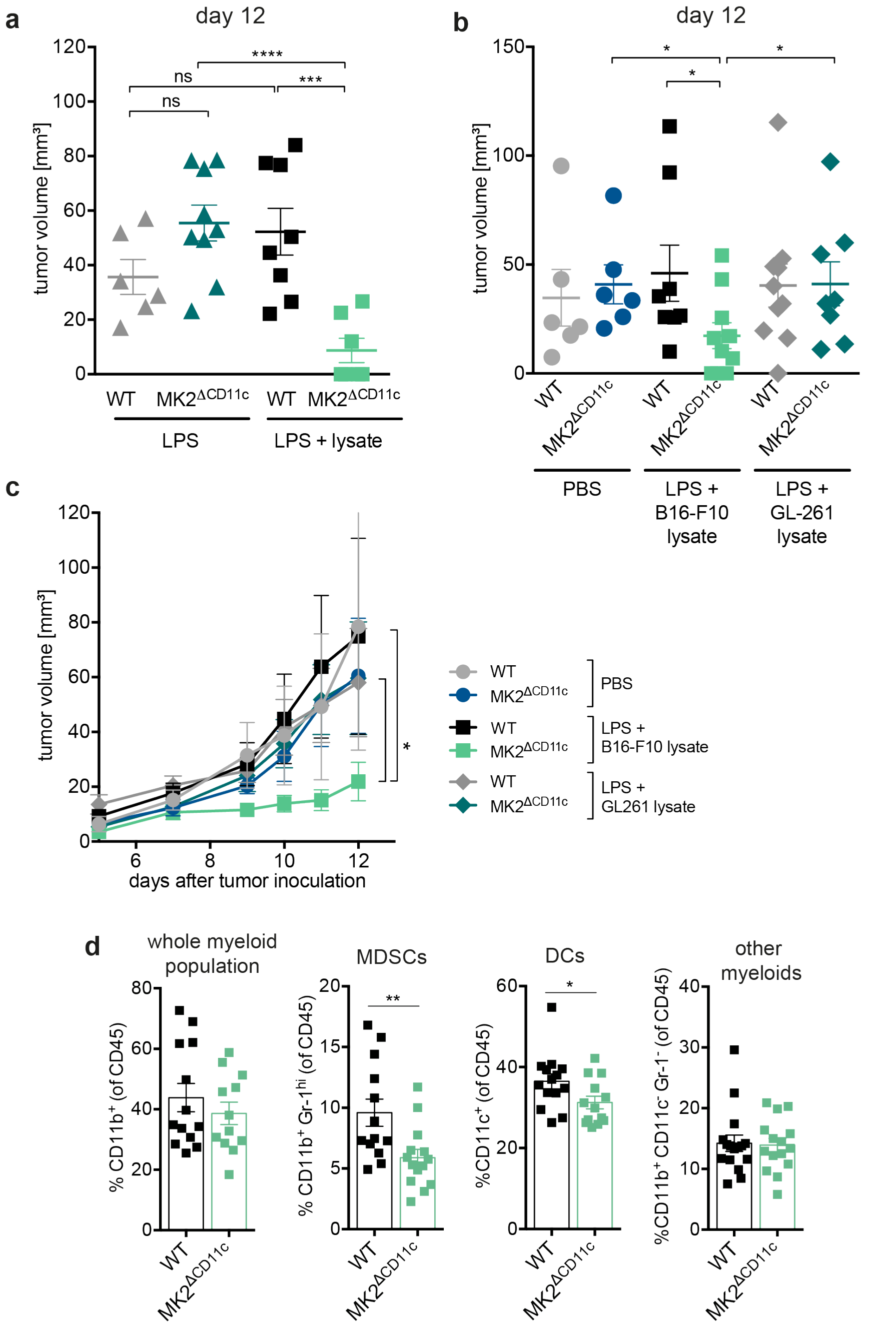

Supplementary Figure 1. Tumours of immunized MK2$^{\Delta CD11c}$ mice are infiltrated by fewer dendritic and immature myeloid cells. (**a-b**) B16-F10 tumour volumes at day 12 post-tumour cell injection in MK2$^{\Delta CD11c}$ and WT control mice immunized with (**a**) LPS alone or in combination with B16-F10 whole cell lysate or (**b**) PBS, LPS + B16-F10 lysate or LPS + GL-261 lysate (n = 5-8 per group, one representative of three independent experiments). (**c**) B16-F10 tumour growth in MK2$^{\Delta CD11c}$ and WT mice immunized with PBS, LPS + B16-F10 or LPS + GL-261 lysate on days 5 and 9 post-tumour cell injection (n = 5-8 mice per group). (**d**) Mean frequencies of overall myeloid cells (CD11b$^+$), DCs (CD11c$^+$ MHC-II$^+$), MDSCs (CD11b$^+$ Gr-1$^+$) and other myeloid populations (CD11b$^+$ Gr-1$^-$ CD11c$^-$) within tumour-infiltrating CD45$^+$ leukocytes measured by flow cytometry. Each symbol represents one individual animal (n = 15-18 per group, pooled from three independent experiments). Data are presented as mean ± SEM. ns, not significant. *P<0.05, **P<0.01, ***P<0.001, ****P<0.0001. P-values were determined using (**a-b, d**) Student's t-test and (**c**) repeated-measures two-way ANOVA with Bonferroni correction for multiple comparisons.

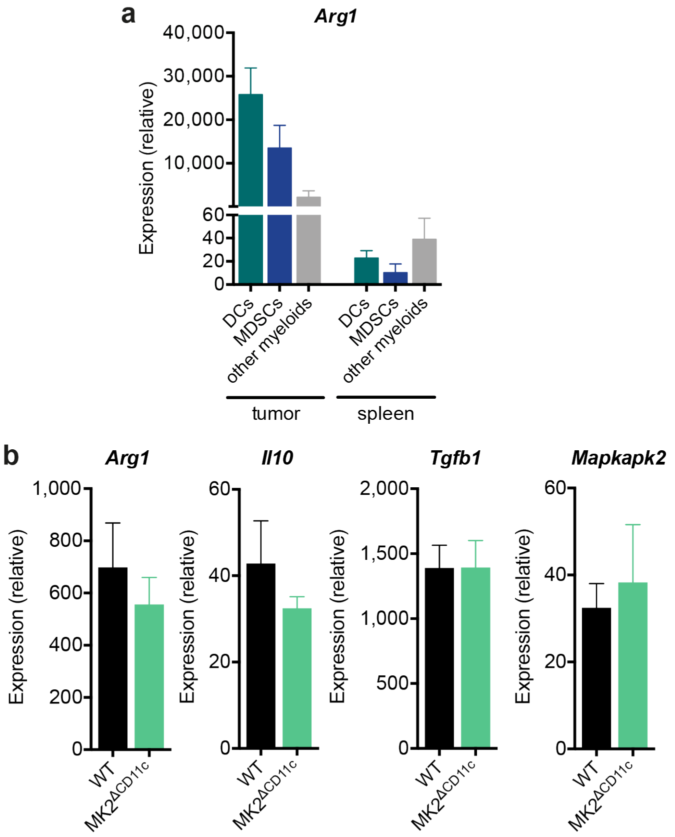

Supplementary Figure 2. Expression of immune suppressive genes in MDSCs is not affected by CD11c-targeted MK2 deletion. (**a**) Relative expression of *Arg1* in myeloid populations sorted from tumours and spleens of melanoma-bearing C57BL/6 WT mice determined by RT-qPCR and normalized to *Ubc* (n = 3). (**b**) Relative expression of *Arg1*, *Il10*, *Tgfb1* and *Mapkapk2* in MDSCs sorted from tumours of $MK2^{\Delta CD11c}$ and WT mice determined by RT-qPCR and normalized to *Ubc* (n = 4). Data are presented as mean ± SEM. *P*-values were determined using Mann-Whitney U test (not significant).

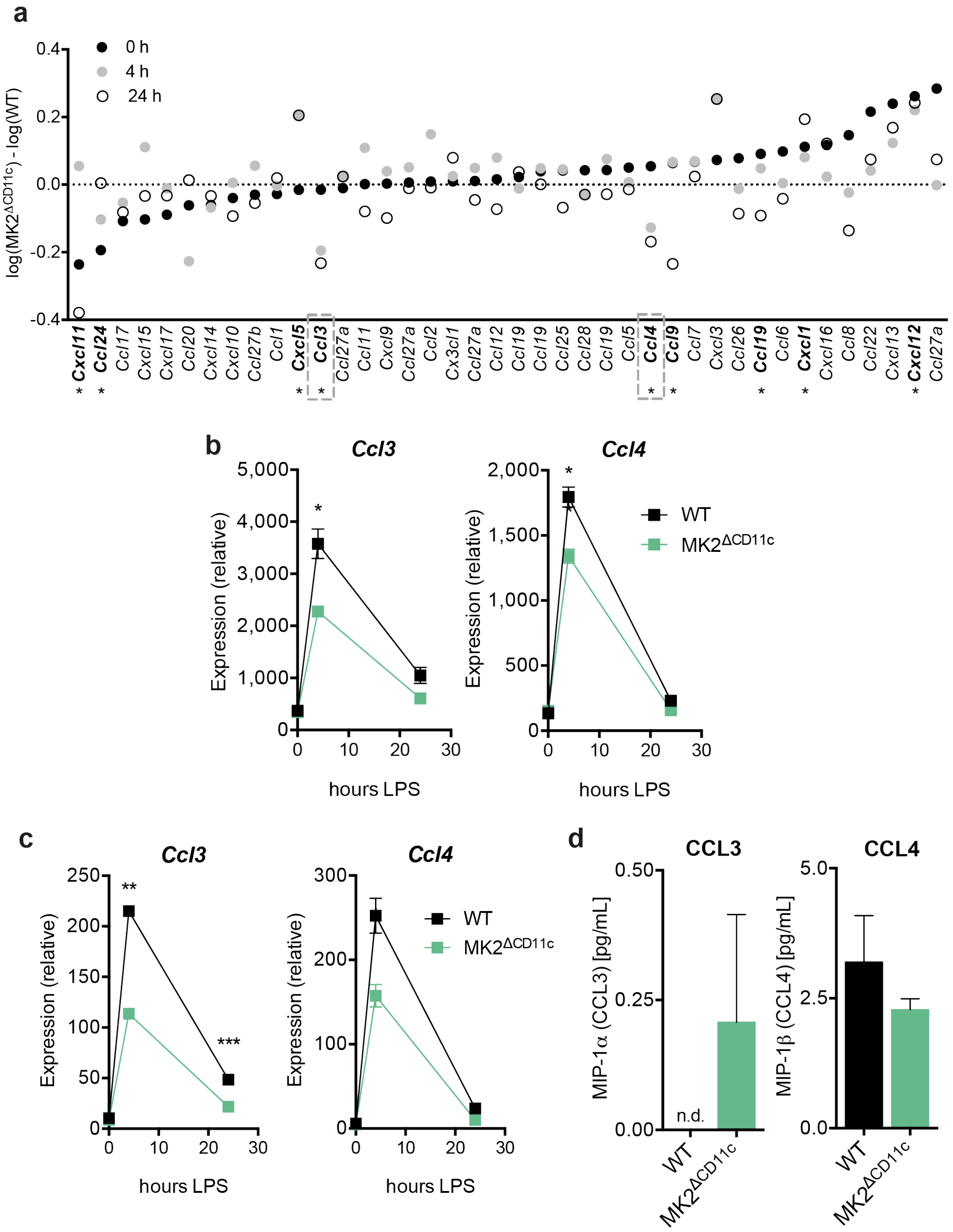

Supplementary Figure 3. Expression of various chemokines is differentially regulated in MK2-deficient DCs. (**a**) Expression of chemokines in splenic CD11c^{+} DCs of MK2$^{\Delta CD11c}$ and WT control mice stimulated over 24 hours with LPS by gene expression array. Differential normalized log2 gene expression between genotypes at 0 (black circles), 4 (grey circles) and 24 hours (white circles) post-LPS stimulation is shown (n = 2). Genes printed in bold show significantly different expression levels between genotypes. (**b-c**) Kinetics of *Ccl3* and *Ccl4* expression in splenic CD11c^{+} DCs over 24 hours of LPS stimulation by (**b**) gene expression array and (**c**) RT-qPCR normalized to *Ubc* (n = 2). (**d**) Serum levels of CCL3 and CCL4 in melanoma-bearing MK2$^{\Delta CD11c}$ and WT mice measured by ProcartaPlex™ Multiplex Immunoassay (n = 5). Data are presented as mean ± SEM. n.d., not detectable. *P<0.05, **P<0.01, ***P<0.001. P-values were determined using (**a-b, d**) Student's t-test and (**c**) Mann-Whitney U test.

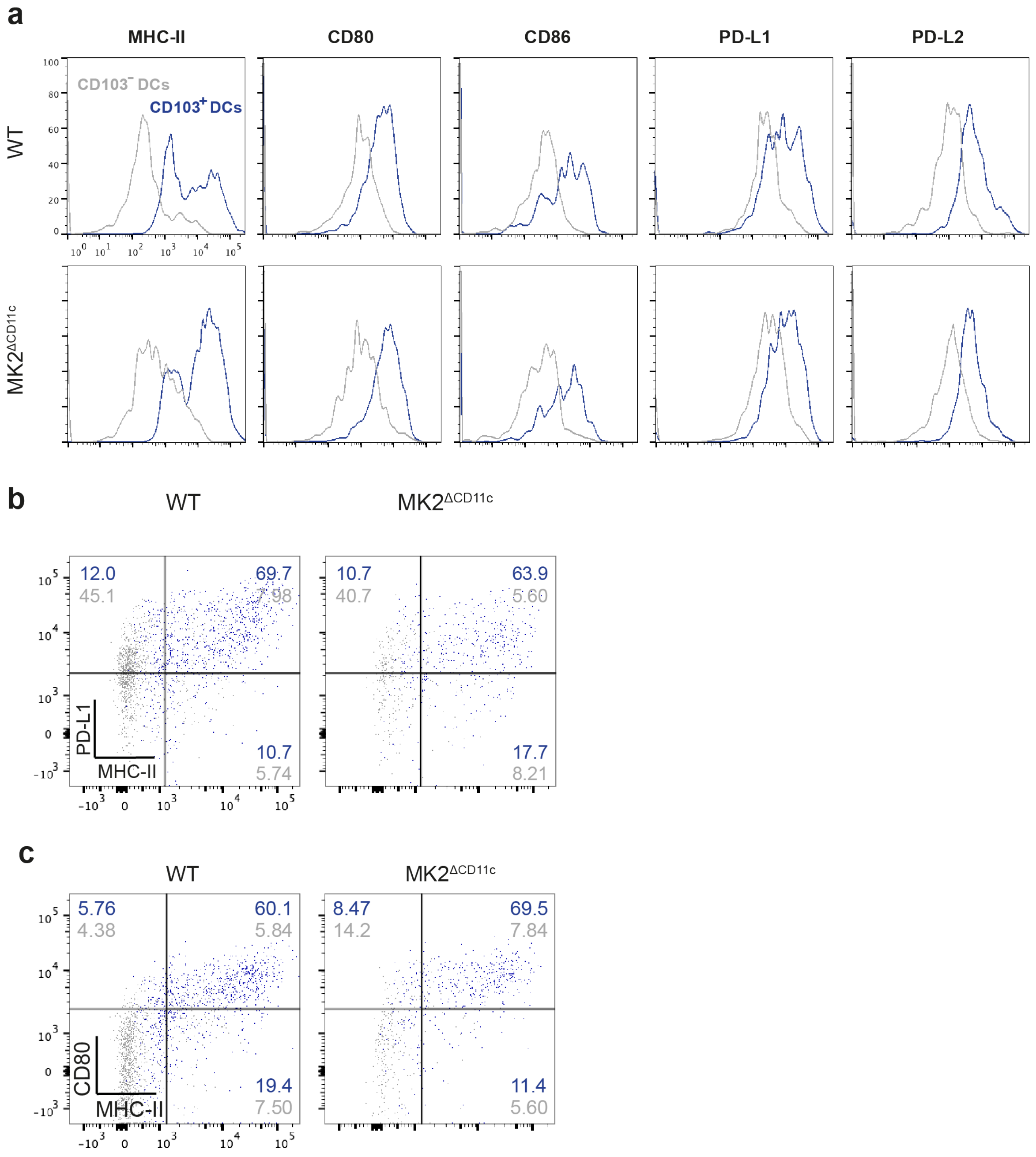

Supplementary Figure 4. CD103⁺ DCs in melanoma-bearing MK2$^{\Delta CD11c}$ mice exhibit a more stimulatory profile. (**a**) Representative flow cytometry histograms showing surface expression of MHC-II, CD80, CD86, PD-L1 and PD-L2 on tumour-resident CD103⁺ (blue) vs CD103⁻ (grey) DCs. Cells were pre-gated for live, single, CD45⁺, CD11c⁺ CD103⁺ or CD103⁻ cells. (**b-c**) Representative dot plots depicting frequency of (**b**) MHC-II- and CD80-expressing DC subpopulations and (**c**) MHC-II- and PD-L1-expressing DC subsets. Frequency within parental population is indicated (blue, CD103⁺ DCs; grey, CD103⁻ DCs).

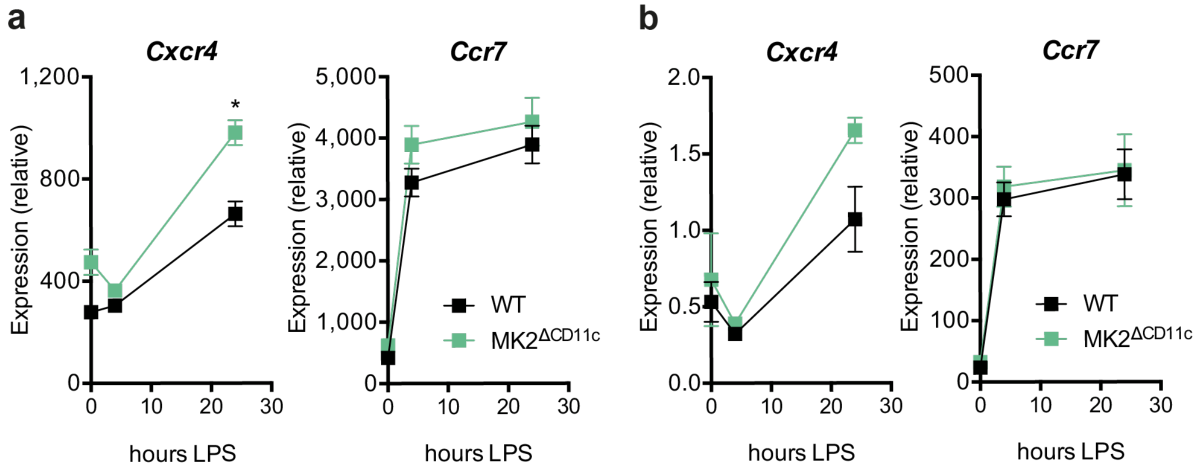

Supplementary Figure 5. ***Cxcr4*** **and** ***Ccr7*** **expression is enhanced upon MK2 deletion in DCs.** (**a-b**) Kinetics of *Ccr7* and *Cxcr4* expression in splenic $CD11c^+$ DCs over 24 hours of LPS stimulation by (**a**) gene expression array and (**b**) RT-qPCR normalized to *Ubc* (n = 2). Data are presented as mean ± SEM. *$P<0.05$. *P*-values were determined using (**a**) Student's *t*-test and (**b**) Mann-Whitney U test.

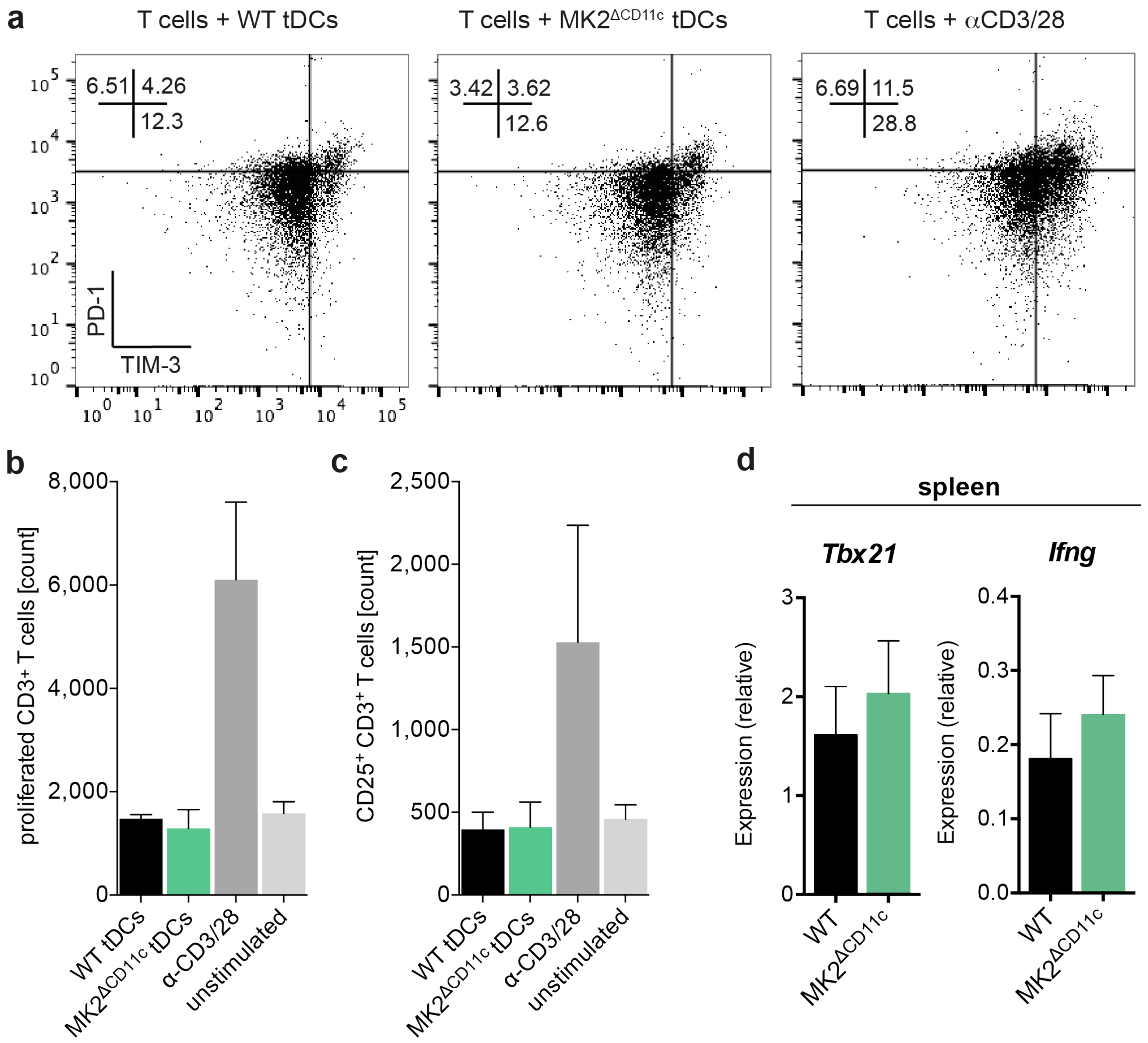

Supplementary Figure 6. CD11c-specific MK2 deletion renders T cells less exhausted and enhances Th1 polarization. (**a**) Representative dot plots depicting relative frequencies of PD-1- and TIM-3-expressing T cells after 5 days of co-culture with freshly isolated $CD11c^{+}$ DCs from tumours of $MK2^{\Delta CD11c}$ and WT control mice. T cells stimulated with αCD3/28 Dynabeads served as activation control. Numbers indicate frequency within parental $CD3^{+}$ population (n = 3). (**b**) Numbers of proliferated T cells after co-culture as analysed by flow cytometry (n = 3). (**c**) Numbers of $CD25^{+}$ T cells after co-culture as analysed by flow cytometry (n = 3). (**d**) *Tbx21* and *Ifng* expression in frozen spleen tissue of tumour-bearing $MK2^{\Delta CD11c}$ and WT littermate control mice by RT-qPCR and normalized to *Ubc* (n = 10-15). Data are presented as mean ± SEM. tDC, tumour-infiltrating DC. *P*-values were determined using (**b-c**) Student's *t*-test and (**d**) Mann-Whitney U test (not significant).

Rearrangement	Productive Frequency (total)	Present in (samples)	Productive Frequency (MK2ΔCD11c)						Productive Frequency (WT)					
			KO1	KO2	KO3	KO4	KO5	KO6	WT1	WT2	WT3	WT4	WT5	WT6
CTTT...GTAC	1.334	4	0.000	0.000	0.000	0.200	0.094	0.000	0.000	0.993	0.000	0.047	0.000	0.000
TCTA...GTAT	1.295	2	0.000	0.000	0.000	0.798	0.000	0.000	0.000	0.496	0.000	0.000	0.000	0.000
TCAA...CTTC	1.505	2	1.310	0.000	0.000	0.000	0.000	0.000	0.000	0.000	0.000	0.000	0.195	0.000
GAGA...GTAT	12.543	2	0.000	0.000	0.000	0.000	0.000	0.000	0.000	0.000	0.000	11.954	0.000	0.588
GACC...CTAT	0.481	2	0.291	0.000	0.000	0.000	0.000	0.000	0.000	0.000	0.000	0.190	0.000	0.000
CATA...CTAT	1.532	2	0.291	0.000	0.000	0.000	0.000	0.000	0.000	1.241	0.000	0.000	0.000	0.000
CACT...CTAC	1.127	2	0.146	0.000	0.982	0.000	0.000	0.000	0.000	0.000	0.000	0.000	0.000	0.000
ATTC...GTTC	0.735	2	0.000	0.000	0.000	0.000	0.000	0.000	0.501	0.000	0.234	0.000	0.000	0.000

Supplementary Table 1. Frequency of T cell receptor (TCR) clones occurring in more than one sample.
